# The Evaluation of Cellulose from Agricultural Waste as a Polymer for the Controlled Release of Ibuprofen Through the Formulation of Multilayer Tablets

**DOI:** 10.3390/bioengineering12080838

**Published:** 2025-08-01

**Authors:** David Sango-Parco, Lizbeth Zamora-Mendoza, Yuliana Valdiviezo-Cuenca, Camilo Zamora-Ledezma, Si Amar Dahoumane, Floralba López, Frank Alexis

**Affiliations:** 1 Departamento de Ingeniería en Biotecnología, Colegio de Ciencias Biológicas y Ambientales (COCIBA), Universidad San Francisco de Quito (USFQ), Quito 170901, Ecuador; 2 Departamento de Ingeniería Química, Colegio de Ciencias e Ingenierías, Universidad San Francisco de Quito (USFQ), Quito 170901, Ecuadorvaldiviezoyuliana2@gmail.com (Y.V.-C.); 3 Bioengineering & Regenerative Medicine Research Group (Bio-ReM), Escuela de Ingeniería, Arquitectura y Diseño (EIAD), Universidad Alfonso X el Sabio (UAX), Avenida de la Universidad 1, Villanueva de la Cañada, 28691 Madrid, Spain; camilza@uax.es; 4 Department of Chemistry and Biochemistry, Université de Moncton, 18 Avenue Antonine-Maillet, Moncton, NB E1A 3E9, Canada; si.amar.dahoumane@umoncton.ca; 5 CATS Research Group, School of Chemical Sciences Engineering, Yachay Tech University, Urcuquí 100119, Ecuador; flopez@yachaytech.edu.ec

**Keywords:** cellulose, controlled release, multilayer tablets, ibuprofen, drug delivery, zero-order kinetics

## Abstract

This research demonstrates the potential of plant waste cellulose as a remarkable biomaterial for multilayer tablet formulation. Rice husks (RC) and orange peels (OC) were used as cellulose sources and characterized for a comparison with commercial cellulose. The FTIR characterization shows minimal differences in their chemical components, making them equivalent for compression into tablets containing ibuprofen. TGA measurements indicate that the RC is slightly better for multilayer formulations due to its favorable degradation profile. This is corroborated by an XRD analysis that reveals its higher crystalline fraction (~55%). The use of a heat press at combined high pressures and temperatures allows the layer-by-layer tablet formulation of ibuprofen, taken as a model drug. Additionally, this study compares the release profile of three types of tablets compressed with cellulose: mixed (MIX), two-layer (BL), and three-layer (TL). The MIX tablet shows a profile like that of conventional ibuprofen tablets. Although both BL and TL tablets significantly reduce their release percentage in the first hours, the TL ones have proven to be better in the long run. In fact, formulations made of extracted cellulose sandwiching ibuprofen display a zero-order release profile and prolonged release since the drug release amounts to ~70% after 120 h. This makes the TL formulations ideal for maintaining the therapeutic effect of the drug and improving patients’ wellbeing and compliance while reducing adverse effects.

## 1. Introduction

Agribusiness generates large amounts of solid and organic biomass residues with a high content of lignocellulosic material [1]. The reuse of these residues has primarily focused on their use as livestock feed, fodder, and compost and in biofuel production [2]. However, these applications may be insufficient due to the growth of agricultural production, which has tripled over the past 50 years [3]. Consequently, much of the agro-industrial waste is disposed of by uncontrolled burning or is dumped into the environment [4]. This highlights the need to develop strategies that reduce the environmental impact of the agro-industry by taking advantage of its waste in various industrial and scientific applications.

Plants’ biomass is mainly composed of cellulose, hemicellulose, lignin, pectin, extracts, and inorganic compounds [5]. Cellulose, a widely available biomaterial made of a linear polymer of glucopyranose linked by β-1,4-glycosidic bonds, represents 40–60% of the biomass [6,7]. Its extraction from agricultural residues offers multiple advantages, such as a low cost, a reduced environmental impact, low energy consumption, and waste reutilization [8]. In addition to its outstanding mechanical properties, this biopolymer is renewable, biodegradable, biocompatible, and non-toxic [9,10].

Cellulose is used in tablet formulations due to its properties as an emulsifier, binder, diluent, disintegrant, thickener, and anti-adhesive [11]. These characteristics make it a key component in drug delivery studies [12], reinforcement composites [13,14,15,16], hydrogels [17,18,19], manufactured coatings [20], matrices [21], tablets [22], and drug films [22]. One of the main current approaches consists of the use of cellulose in sustained drug release systems, which aim to control the release of the active ingredient to improve its efficacy [23] and avoid abrupt fluctuations of the drug concentration in the body [24].

Multilayer tablets consist of a core with the drug surrounded by one or more impermeable or semi-permeable barriers that regulate the release of the active ingredients [25,26,27,28,29,30,31]. These formulations can maintain stable therapeutic concentrations for a longer time and generate zero-order release profiles, thus ensuring a steady, constant release [32,33]. In addition, they offer some advantages, such as preventing/minimizing the degradation of active ingredients, combining several drugs in the same tablet, acting as a buffer for unstable ingredients, and a suitability for high-dose drugs [26,32,34].

Ibuprofen is taken as a model drug to evaluate these multilayer tablets. This is a non-steroidal anti-inflammatory drug (NSAID) with antipyretic and analgesic effects [35]. However, its prolonged consumption is associated with adverse effects on the gastrointestinal tract, a risk of ulcers, renal damage, and alterations in blood coagulation [36]. It is estimated that one in five chronic users will develop gastric damage; in consequence, it is recommended to limit its intake to low doses and short periods [37]. Commercial ibuprofen tablets are fast-release with a rapid decrease in their effect [38], which represents a disadvantage in the management of chronic diseases, such as osteoarthritis and rheumatoid arthritis, where frequent administration is required (every 4 to 6 h), therefore increasing the risk of side effects [39].

Ibuprofen’s release from different formulations depends on various factors, such as the drug solubility [40], characteristics of the polymeric matrix, and dissolution medium conditions [41]. Studies have demonstrated that the dissolution rate of ibuprofen can vary significantly depending on the dissolution apparatus and medium pH. For instance, when comparing 400 and 600 mg ibuprofen tablets under various conditions, it is observed that both the dosage and dissolution system significantly influence the rate and extent of the drug release [41].

Polymeric matrices control the ibuprofen release primarily through diffusion and erosion mechanisms. The selection of the polymer type and its interaction with the drug is crucial to determine the drug release profile [42]. For example, sustained-release formulations incorporating xanthan gum, polyethylene glycol (PEG), and polycaprolactone (PCL) have been developed, achieving controlled and prolonged ibuprofen release [43].

In this context, cellulose extracted from plant waste could be used as a matrix for the development of controlled-release ibuprofen tablets using a multilayer system. Therefore, this research focuses on the extraction, processing, and characterization of cellulose from agricultural residues and the evaluation of their effect in controlled release studies of ibuprofen formulated as multilayer tablets.

## 2. Materials and Methods

### 2.1. Materials

Cellulose was extracted from two sources: orange peel (*Citrus sinensis* L. Osbeck) and rice husk (*Oryza sativa*), referred to as OC and RC, respectively, biomass was acquired in the Cumbaya market (Quito, Ecuador). A commercial cellulose (CC) sample was used as a reference, specifically “Sigmacell Cellulose: Type 101, Highly Purified, Fibers” purchased from Sigma-Aldrich (Darmstadt, Germany).

Ecuagen commercial brand of 800 mg ibuprofen was used. The coating from the commercial tablet was removed, and the tablet content was ground and used as is with all its excipients.

### 2.2. Cellulose Extraction

The biomass was first boiled in water at a 1:1 ratio. The chemical extraction of cellulose was carried out based on an acid/base protocol, then bleached and washed with water until chemical by-products were removed [7,44,45]. This process was carried out under the same conditions for OC and RC types of cellulose.

### 2.3. Physicochemical Characterization

#### 2.3.1. Fourier Transform Infrared Spectroscopy

To identify the chemical groups present, cellulose samples were characterized by Fourier transform infrared spectroscopy (FTIR) in a Cary 630 FTIR Spectrometer (Agilent Technologies, CA, USA). The determined range was from 3400 to 600 cm^−1^. The chemical structure of the different samples obtained was evaluated. The changes undergone by the functional group bands of cellulose extracted from rice husks and orange peels provide information about the purity of the material extracted. For a convenient analysis, all the spectra were normalized with respect to the most intense peak.

#### 2.3.2. Thermogravimetric Analysis (TGA)

A thermal analysis technique is used to evaluate the thermal stability and decomposition properties of extracted materials by measuring the weight changes in the sample when the temperature increases in a controlled atmosphere [46]. TGA is normally performed at a constant heating rate of 10 °C/min, starting at room temperature and progressing up to approximately 1000 °C. The temperature range to be used must be defined according to the nature of the material. During the analysis, three main stages are usually observed: release of volatiles and adsorbed water, degradation of the material, and combustion of the organic fraction.

To evaluate the thermal stability of the three types of cellulose (OC, RC, and CC), a thermogravimetric analyzer (TGA) 55 Discovery 0550-1517 equipment (TA Instruments- Waters Corporation, New Castle, DE, USA) in the temperature range of 25–550 °C was used with a heating rate of 10 °C/min under a nitrogen atmosphere.

#### 2.3.3. X-Ray Diffraction (XRD)

An X-ray diffraction (XRD) analysis was conducted to investigate the crystal structure of cellulose extracted from orange peel and rice husk. Powdered samples were placed on a flat sample holder to ensure a homogeneous and smooth surface, essential for accurate diffraction measurements. X-ray diffraction (XRD) pattern data were recorded using a Miniflex-600 diffractometer (Rigaku Corporation, Akishima, Tokyo, Japan) equipped with a D/tex Ultra2 detector; the X-ray generator was operated at 40 kV and 15 mA, using a sealed tube CuKα radiation source. For data collection in reflection mode, a θ/2θ scan axis was employed with a 0.01° step width in a scan range of 5–90° in a 2θ mode, and the D/tex Ultra2 detector in 1D scan mode. Furthermore, a Soller slit of 1.25° was used for received and incident scattering, with high-length, receiving, and incident slits of 10.0 mm, 8.0 mm, and 13.0 mm, respectively. In addition, the recorded XRD data were treated using the SmartLab Studio II program.

The crystallinity index for each sample was determined using the Segal equation:

$$CI\left(\%\right)=\frac{I_{002}-I_{am}}{I_{002}}\times 100$$

where I_002_: maximum intensity of the peak corresponding to the crystalline plane. I_am_: minimum intensity between the (002) and (101) peaks that represent the amorphous fraction. 

### 2.4. Tablet Preparation

Cellulose and ibuprofen were compressed in a pressure- and temperature-resistant mold. For this, five formulations were established for each type of cellulose: cellulose (C) as the negative control, compressed commercial ibuprofen (IB) as the positive control, cellulose mixed with ibuprofen (MIX), cellulose and ibuprofen in bilayer (BL), and cellulose and ibuprofen in trilayer (TL). Table 1 details the composition for each type of tablet and type of cellulose. Tablets fabricated (1.25 cm height × 1.00 cm diameter) with defects and loss of material were discarded to maintain consistent dimensions and weight for all tablets used in this study.

A heat press was used for compression. For each tablet type, specific temperatures and pressures were used. Additionally, the number of compressions was performed considering the number of layers according to previously reported protocols (Table 2) [32,47].

For BL and TL tablets, each temperature belongs to a number of compressions. That is, the first temperature corresponds to the first compression and vice versa. After compression, the produced tablet was allowed to cool down to room temperature and stored in the freezer without any further treatment.

### 2.5. Hardness of Tablets

A digital Shore D durometer was used (Model MILA037A00; Guangzhou Landtek Instruments Co. Ltd., Guangzhou, Guangdong, China) to study the hardness of commercial ibuprofen tablets vs. lab-made analogs based on OC, RC, and CC. The average of 5 measurements throughout one sample was reported. According to the ASTM D2240 standard [48], indentation force (*N*) can be obtained through the equation:

*N* = (0.443)(*HD*) [*N*]

where *N* is the indentation force in Newtons, and *HD* is the hardness value measured by the instrument.

### 2.6. Ibuprofen Release and Quantification

Ibuprofen release was studied in triplicate in phosphate-buffered saline (PBS) medium at pH 7.4 [49]. The tablets were used in triplicate and placed in Falcon tubes containing 100 mL of PBS and incubated with their cap on at 37 °C for 120 h. Then, 1.5 mL aliquots were taken at the periods of 1, 2, 3, 6, 24, 48, and 120 h. After each aliquot was obtained, the same volume of PBS was replenished. These samples were refrigerated for subsequent analysis. The calibration curve was prepared using concentrations from 5 to 42 µg/mL with R^2^, the coefficient of linearity, of 0.9986. The detection limit was about 3 µg/mL.

To quantify ibuprofen, a calibration curve for ibuprofen was established using a HANON i5 UV-VIS spectrophotometer (Hanon Instruments, Jinan, China). The maximum detection peak absorbance was determined at 222 nm. The absorbance of the aliquots was measured, and serial dilutions were performed if the measured absorbance was greater than one absorbance unit (AU).

The percentage of ibuprofen release overtime was determined using the following formula:

$$R_{IB}\left(\%\right)=\frac{m(t)}{m(total)}\times 100$$

where $R_{IB}\left(\%\right)$ indicates ibuprofen release, m(t) is the mass accumulated over time, and m(total) is the total initial mass [50].

## 3. Results

A chemical extraction process of the cellulose from the orange peel (*C. sinensis* L. Osbeck) and rice husk (*O. sativa*) waste was carried out. The as-obtained, unmodified cellulose fibers were further characterized and used to design multilayer tablets to release ibuprofen. For comparison purposes, commercial cellulose (CC) was used.

### 3.1. Functional Groups of Commercial Cellulose

**Figure 1:**
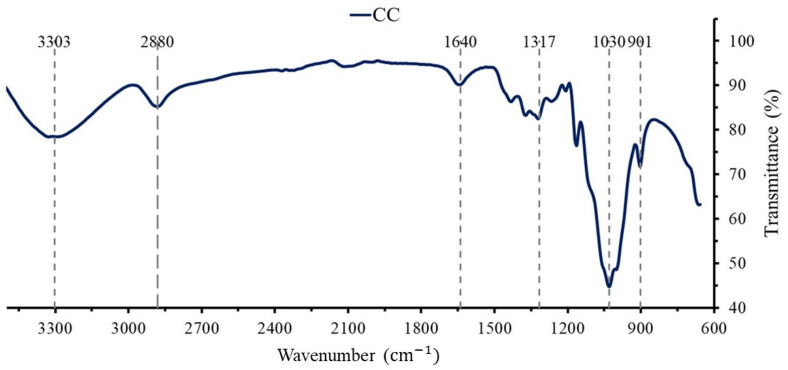
FTIR pattern of commercial cellulose (CC).

The FTIR spectrum of CC (Figure 1) exhibits the characteristic chemical groups of cellulose, such as C-C at 901 cm^−1^, C-OH at 3303 cm^−1^, C-H at 1317 cm^−1^, and a C-O ring at 1030 cm^−1^ in addition to side vibration bands at ∼901 cm^−1^ and ∼1317 cm^−1^. The glycosidic ether bond C-O-C in the pyranose ring appears at ∼1030 cm^−1^. Other noticeable peaks are the vibration of water molecules in cellulose at ∼1640 cm^−1^, the C-H stretching vibrations of hydrocarbon at ∼2880 cm^−1^, and the stretching vibration of the hydroxyl groups in polysaccharides (O-H) at ∼3303 cm^−1^ [7,51].

### 3.2. Functional Groups of Agricultural Waste Extracted Cellulose

**Figure 2:**
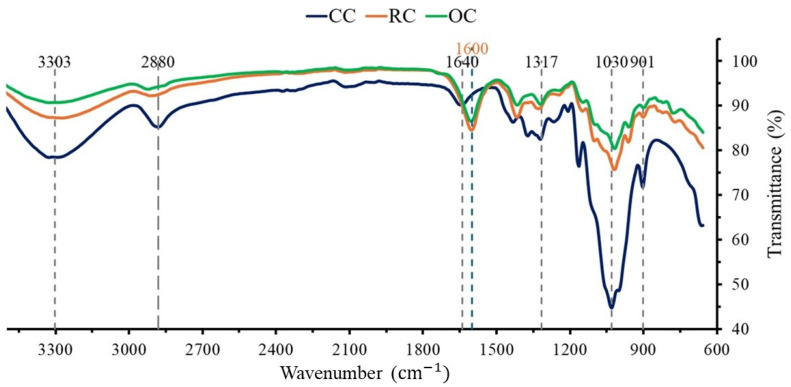
FTIR spectra of waste-extracted cellulose from rice husk (RC, in orange) and orange peel (OC, in green), compared with commercial cellulose (CC, in blue). RC and OC samples show the same bands at the same wavenumbers. They share this feature with most CC bands (in black). However, the CC band located at 1640 cm^–1^ (in blue) shifts to 1600 cm^–1^ (in orange) owing to water presence in large amounts in agrowaste-extracted cellulose. This constitutes the main difference between CC and lab-extracted cellulose.

Cellulose samples extracted from the orange peel and rice husk show no significant differences with CC in terms of functional groups, since mainly similar characteristic chemical groups present in CC are identified (Figure 2). However, slight differences were found at ∼1600 and ∼1640 cm^−1^; this may be attributed to the vibration of water molecules in the agricultural cellulose due to more bending of the OH groups in their structure, suggesting a greater water binding than in its commercial counterpart.

### 3.3. Thermal Stability of Cellulose 

**Figure 3:**
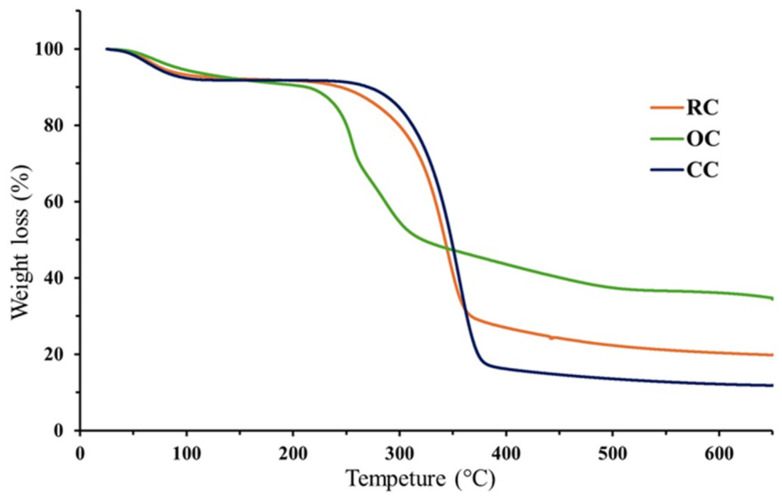
Comparative TGA of cellulose extracted from rice husk (RC) and orange peel (OC) with its commercial counterpart (CC).

The thermogravimetric analysis (TGA) of cellulose typically reveals multiple stages of mass loss associated with the decomposition of its main structural components as the temperature increases, as shown in Figure 3. Initially, moisture loss occurs between 25 and 125 °C (<10% of mass loss), attributed to the evaporation of the physically adsorbed water [52]. Between 175 and 225 °C, hemicellulose begins to degrade, mainly through the breakdown of carbohydrates and pectin [53]. In all cases, this represents a tiny percentage of mass loss for the RC and CC, while it amounts to ~15% for the OC. The substantial mass loss corresponding to the cellulose degradation is commonly observed between 290 and 375 °C. This accounts for a ~75% mass loss for CC, a ~60% mass loss for the OC, and a ~35% mass loss for the RC. Above 385 °C, an additional weight reduction is generally linked to the thermal degradation of the residual lignin.

Based on these characteristic thermal transitions, a comparative analysis of the RC, OC, and CC samples was conducted. The commercial cellulose (CC) sample served as the standard reference to assess the thermal stability and structural purity of cellulose extracted from rice (RC) and orange (OC) residues. This study forms part of a broader investigation aimed at evaluating agricultural waste-derived cellulose as functional biopolymers in multilayer tablet formulations for the controlled release of ibuprofen. TGA results show that the CC sample exhibits the highest mass loss related to the cellulose fraction (65%), with a major degradation peak at 371 °C, indicating a high degree of purity. In contrast, the OC sample has a significantly higher final residue (34.7%), suggesting a greater content of lignin and/or other non-cellulosic components, while the RC shows an intermediate behavior with a residue of 19.8%.

Moreover, the onset of the hemicellulose degradation occurs at lower temperatures in RC (~210 °C), followed by OC (~235 °C) and CC (~288 °C), indicating differences in the structural composition and purity levels among the samples. Although CC exhibits a superior thermal performance, both RC and OC samples demonstrate an adequate thermal stability suitable for pharmaceutical processing. These findings support their potential use as matrix-forming agents in controlled-release drug delivery systems. In particular, the RC sample, owing to its low residue content and favorable thermal degradation profile, appears promising for the incorporation into ibuprofen-loaded multilayer tablet formulations.

### 3.4. X-Ray Diffraction Analysis (XRD)

**Figure 4:**
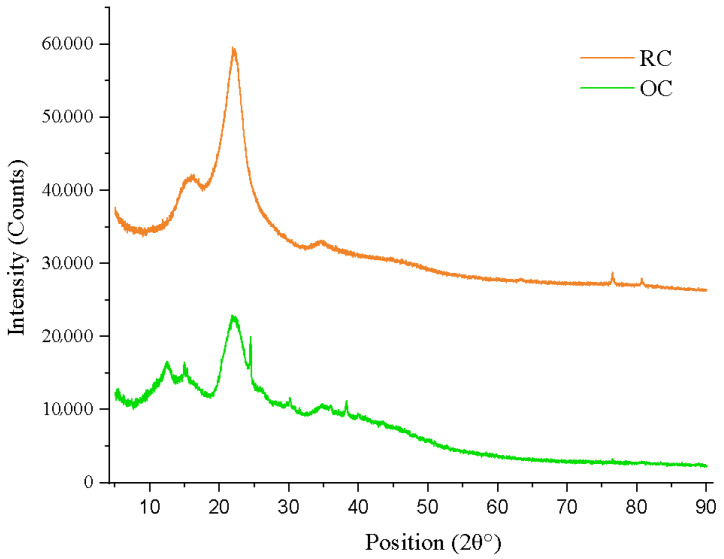
Comparative XRD analysis of cellulose extracted from rice husk (RC) and orange peel (OC).

The X-ray diffraction analysis performed on cellulose samples extracted from rice (RC) and orange (OC) residues reveals structural features characteristic of type I cellulose, indicating a semicrystalline nature of both materials (Figure 4) [54]. The diffractograms show well-defined peaks within the 2θ range of 14 to 24°, typically associated with the ($\bar{11}0$), (110), and (200) crystalline planes of cellulose I [55]. In particular, a prominent peak was observed at 2θ ≈ 22.5°, corresponding to the (200) plane, with an interplanar spacing (*d*-spacing) of approximately 0.395 nm, calculated using Bragg’s law and a Cu-Kα radiation wavelength of λ = 1.5406 Å. Based on this data, the crystallinity index (CI) was calculated, yielding values of 48.7% for the OC sample and 55.5% for the RC one. These results indicate that rice-derived cellulose exhibits a higher crystalline fraction, likely due to a lower proportion of amorphous regions and a more ordered molecular structure.

### 3.5. The Hardness of the Tablets

To better understand the characteristics of the tablets, the commercial ibuprofen was established as a hardness reference. The hardness (HD) of the tablets was measured in four different regions of each one, the upper face, lower face, front side, and back side, using Durometer PCE-DD-A Shore A. The values obtained were averaged to determine the overall hardness of each tablet type on a 0–100 HD scale (Table 3) [56]. As a result, the hardness of the lab-made RC and OC tablets remains close to that of the reference cellulose and commercial ibuprofen tablets, highlighting the suitability of the cellulose recovered from agrowaste in the design of oral drug delivery systems [57].

### 3.6. Ibuprofen Release Profiles

#### 3.6.1. Ibuprofen Release from Tablets Made of Commercial Cellulose

**Figure 5:**
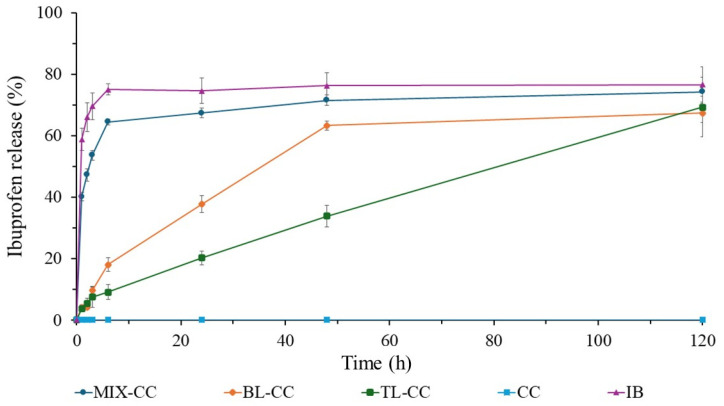
Ibuprofen (IB) release profiles of the commercial cellulose (CC, the negative control), commercial IB tablet (the positive control), and different tablets made of CC and IB compressed into different formulations: mixing IB with CC (MIX-CC), a layer of CC and a layer of IB (BL-CC), and two layers of CC sandwiching IB (TL-CC).

Figure 5 shows the ibuprofen release profile of different formulations made of commercial cellulose, indicating that the position of the cellulose during the tablet compression directly influences the release rate and release profile in PBS at pH 7.4.

The commercial ibuprofen tablet (IB) shows an accelerated release profile since more than 70% of the drug is released within the first 6 h. Then, this process seems to plateau at ~80% for the rest of the study time. It is therefore the ideal positive control for comparison with multilayer formulations. On the other hand, the tablet made of only commercial cellulose (CC) does not show any release and, for this reason, is the suitable negative control. The tablet produced by mixing ibuprofen with commercial cellulose (MIX-CC) shows a slight delay in drug release during the first 6 h, as it exceeds the 60% release. Afterwards, its release profile approaches that of IB and plateaus at almost the same percentage (~80%). Overall, the MIX-CC profile resembles that of a commercial ibuprofen tablet.

The tablet made of commercial cellulose with the drug in two layers (BL-CC) presents greater control in the release rate and takes longer to achieve ibuprofen dissemination in the medium. In the first 6 h, it exhibits a significantly lower percentage of drug release than the IB and MIX-CC, by approaching 20%. After 6 h, the release profile seems to be linear until 48 h, where it reaches its maximum release peak at ~65%.

In the case of the three-layer tablet (TL-CC), the two cellulose layers sandwiching the ibuprofen directly influence the drug release. For the first 6 h, the drug release reaches hardly 10%. Then, the release rate increases linearly during the 120-h study until it exceeds 60%. It is important to notice that within the study period, this sample does not plateau, unlike the other samples made of CC, suggesting an even longer linear release profile.

#### 3.6.2. Ibuprofen Release from Tablets Made of Rice Husk Cellulose

**Figure 6:**
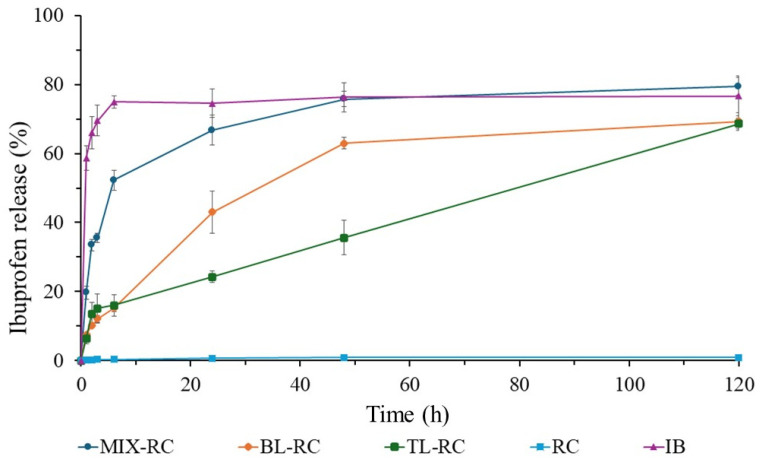
Ibuprofen (IB) release profiles of the rice husk cellulose (RC, the negative control), commercial IB tablet (IB, the positive control), and different tablets made of RC and IB compressed into different formulations: mixing IB with RC (MIX-RC), a layer of RC and a layer of IB (BL-RC), and two layers of RC sandwiching IB (TL-RC).

Figure 6 uses the profile of the commercial ibuprofen (IB) as the positive control, while the rice husk cellulose (RC) becomes the negative control since it does not display any IB release. The mixed tablet made of rice cellulose and ibuprofen (MIX-RC) shows its equivalence to the commercial cellulose (MIX-CC) by delaying the drug release rate. In this case, the drug release exceeds 50% within the first 6 h. However, this rate is still lower than that of the MIX-CC (60%). After 48 h, the MIX-RC drug release plateaus at ~75%.

In the first 6 h, the drug release from the two-layer tablet (BL-RC) is slightly lower than that of the three-layer tablets (TL-RC). At 6 h, both samples show the same drug release rate (~15%). Afterwards, an inversion of drug release profiles occurs as the BL-RC exhibits a faster release profile than the TL-RC. In addition, the BL-RC plateaus at ~62% within 48 h. On the other hand, the TL-RC displays a linear release profile between 6 and 120 h. At 48 h, it exceeds a 30% release and reaches ~62% at the end of the study. Among all RC formulations, the TL-RC is the only one that does not show any tendency to plateau within the study period, indicating that a longer linear release is attainable.

#### 3.6.3. Ibuprofen Release from Tablets Made of Orange Peel Cellulose

**Figure 7:**
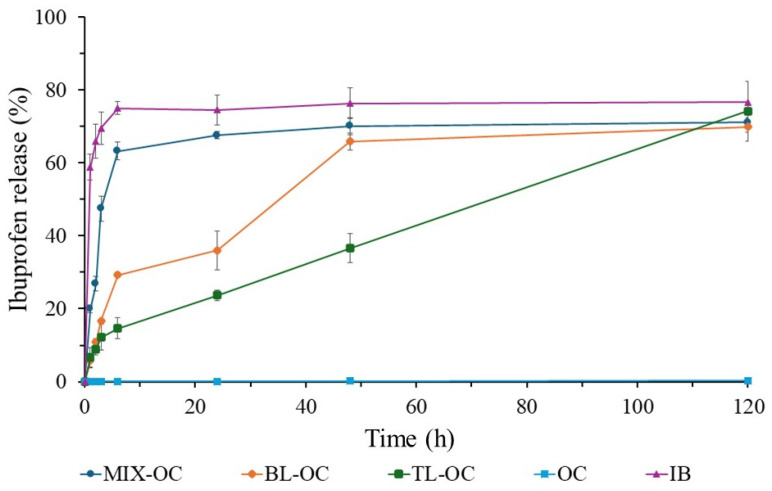
Ibuprofen (IB) release profiles of the orange peel cellulose (OC, the negative control), commercial IB tablet (IB, the positive control), and different tablets made of OC and IB compressed into different formulations: mixing IB with OC (MIX-OC), a layer of OC and a layer of IB (BL-OC), and two layers of RC sandwiching IB (TL-RC).

Figure 7 regroups the ibuprofen release profiles of tablets formulated using the orange peel cellulose (OC). In this case, the commercial IB tablet is kept as the positive control, while the tablet based solely on the OC, which does not show any IB release, is taken as the negative control. Within the first 6 h, the MIX-OC tablet exhibits a release profile that resembles MIX-CC since it releases more than 60% of the loaded IB. Then, the drug release slightly increases to approach 70% at 48 h. Subsequently, the release rate does not increase and finally plateaus.

When compared to TL-OC, the BL-OC displays an overall faster release profile. Within the first 6 h, ~30% of the IB is released from BL-OC vs. the ~15% release from the TL-OC. Then, the drug release exceeds 60% for the BL-OC and tends to plateau afterwards at ~70%. On the other hand, the TL-OC release is linear between 6 and 120 h when it reaches more than 70% of the IB release. In addition, the TL-RC is the only formulation based on OC that does not show any tendency to plateau within the study period, inferring a possible longer zero-order release.

#### 3.6.4. Comparison Between Two-Layer and Three-Layer Release Profiles

**Figure 8:**
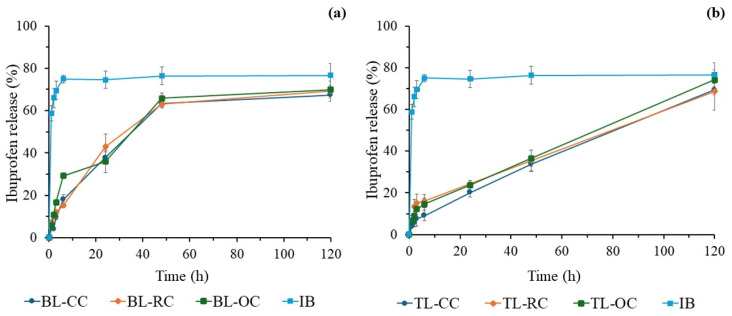
Comparison of ibuprofen release profiles of multilayer IB tablets: (**a**) bilayer tablets made with commercial cellulose (BL-CC), rice husk cellulose (BL-RC), and orange peel cellulose (BL-OC) and (**b**) trilayer tablets made with commercial cellulose (TL-CC), rice husk cellulose (TL-RC), and orange peel cellulose (TL-OC).

The results in Figure 8 compare the profile and release percentage of ibuprofen from bilayer tablets (a) and trilayer tablets (b). Firstly, the IB release profiles of BL and TL tablets clearly differ from those of commercial IB (Figure 8a,b). While the commercial IB tablet shows a fast drug release that reaches its plateau at ~75% within 6 h, the BL tablets display a slower and steady release for the first 48 h before plateauing at 62–65% (Figure 8a). On the other hand, TL tablets exhibit an even slower and linear release for the period spanning from 6 to 120 h without plateauing. When comparing the BL tablets, slight differences in their release profile are observed. For example, the BL-OC tablet shows a higher release percentage during the first 6 h, reaching 30%, while both the BL-CC and BL-RC have percentages below 20%. However, after 24 h, the release percentages and profiles become quasi-identical for all BL tablets regardless of the cellulose type. At 48 h, their IB release exceeds 60% and tends to plateau afterwards at 65–70%.

In the case of the three-layer tablets (Figure 8b), all tablets show a similar release profile and rate. Within the first few hours, the IB release rate remains below 20%, then becomes linear for the 6–120 h period of study to ultimately exceed the 70% release rate, approaching the result of the commercial tablet but, interestingly, without plateauing, suggesting that the release may continue likewise to its full completion for longer periods.

Overall, the recorded data regarding the IB release demonstrate that the three investigated types of cellulose, i.e., commercial, orange peel, and rice husk ones, perform equivalently when compressed into multilayer tablets, either as a bilayer or trilayer, as determined by their IB release profiles that are almost identical for all BL tablets and for all TL analogs too.

## 4. Discussion

Nowadays, biomaterials derived from renewable resources can reduce the environmental impact caused by agro-industrial activities [8]. The use of plant waste resources has been described in various studies not only as a source of polymers but also of microorganisms, enzymes, and chemical compounds [10]. So far, the excipients used in the pharmaceutical industry have been directly obtained from plant species and mineral resources with extraction processes that can release harmful compounds in addition to the energy demand [58]. In this regard, the cellulose extracted from plant waste constitutes a viable and sustainable alternative for controlled release studies of ibuprofen.

Fourier transform infrared spectroscopy (FTIR) is a technique used to characterize molecular structures, allowing the identification of functional groups [51,59]. In Figure 2, no significant differences can be seen between the cellulose extracted from orange and rice wastes and their commercial counterpart. This is important because it guarantees their equivalence in terms of chemical composition, allowing their use as a polymer for the compression of tablets. Since cellulose is extracted from agricultural waste, lignin and hemicellulose must be removed due to the heterogeneity of the initial biomass [4]. The most notable difference between the FTIR of CC with RC and OC is visualized at ∼1600 and ∼1640 cm^−1^, respectively. This is related to the vibrations of water molecules when bending the OH group and may be due to the presence of water in the sample due to the retention of molecules [7,51]. Nevertheless, this difference is not detrimental since an additional dehydration step can remove water molecules in excess [58].

TGA results reveal significant differences in thermal stability and structural composition among the evaluated cellulose samples (Figure 3). The thermal behavior of the CC sample serves as a reference for assessing the purity and functionality of the cellulose extracted from agro-industrial residues. The lower final residue observed in the RC sample suggests a more efficient removal of non-cellulosic components, resulting in a more defined thermal degradation pattern and greater structural similarity to the standard cellulose. In contrast, the OC sample displays a higher mass retention at elevated temperatures, which may indicate the presence of lignin, pectin, and/or other insoluble materials that were not fully eliminated during the extraction process [60]. From a functional standpoint, both types of cellulose demonstrate sufficient thermal stability suitable for pharmaceutical applications; however, the rice-derived cellulose exhibits a more favorable profile for use as a matrix-forming agent in controlled-release systems due to better thermal stability.

The X-ray diffraction (XRD) analysis confirms that both RC and OC samples exhibit a semicrystalline structure corresponding to type I cellulose, as evidenced by the appearance of characteristic peaks with a prominent maximum around 2θ ≈ 22.5°, corresponding to the (200) plane (Figure 4). These findings are consistent with previously reported results that analyzed commercial cellulose used in the fabrication of antibacterial hydrogels and identified similar peaks indicative of typical crystalline regions of the native cellulose [17]. The rice residue appears to be a more structurally ordered source of cellulose, which may be related to a lower presence of impurities and/or a more efficient extraction process that removes amorphous components. On the other hand, the OC sample presents broader and less intense peaks, suggesting a higher proportion of amorphous domains. This behavior may be attributed to the heterogeneity of the plant matrix of the orange residue, which usually contains higher amounts of pectin, hemicellulose, and lignin [55].

The pressure and temperature are essential factors for tablet compression. A high pressure causes a greater penetration between particles with better interactions between layers, which facilitates the adhesion between phases or layers [32]. In the case of cellulose, studies indicate that the combination of high temperatures and pressures improves the mechanical properties of biopolymers or cellulose-based compounds [47,61]. In addition, tablets can be obtained without the use of binders, making the process straightforward. This is because cellulose increases its crystallinity due to pressure to become more compact. The material loses porosity owing to the compression process that causes particle breakage due to internal friction, leading to an increase in the material density when the temperature threshold is reached [27,61]. The tablets visually lose their porosity to become harder and denser than the starting powder material. This induces the formation of polymeric barriers or layers useful for the release system since cellulose acts as a matrix that covers and encapsulates the loaded drug ibuprofen. Once compressed, the tablet gains stability in the environment by maintaining its mass; that is, it does not show signs of erosion since the material’s intermolecular integration has been promoted. The results from Table 3 demonstrate that the hardness of all fabricated tablets and their commercial analog is remarkably similar, meaning that the study of their release kinetics will provide useful insight based on the way they are made and/or their composition rather than on their hardness.

The ibuprofen release in PBS at pH 7.4 varies according to the formulation of the tablets with cellulose. As seen in Figure 5, Figure 6 and Figure 7, the positive ibuprofen control (IB) shows a rapid release during the first 6 h. This is because most commercial ibuprofen formulations are designed for immediate release, with a plasma half-life of approximately 2 to 3 h [37,39]. For this reason, ibuprofen needs to be administered in multiple intakes to maintain its therapeutic effect, which may increase the risk of adverse effects.

For each cellulose type, the negative control consists of tablets compressed exclusively with CC, RC, and OC cellulose. UV-Vis measurements at 222 nm detect no absorbance attributable to ibuprofen, confirming the accuracy of the equipment and the validity of the experimental procedures.

When comparing the release profile of the commercial ibuprofen with cellulose-containing formulations, significant differences in the release rate and profiles are observed. Figure 5, Figure 6 and Figure 7 show that cellulose directly influences the drug release rate by acting as a diffusion barrier. In MIX tablets, cellulose affects the release rate but not as efficiently as it should. Although it delays the release during the first 3 h, cellulose’s uniform distribution in the tablet generates a fast first-order release profile, like that of commercial formulations where the excipient and drug are well mixed [25,62,63].

In bilayer tablets (BL), the cellulose layer serves as a polymeric matrix that retains ibuprofen for a longer period. The drug release occurs mainly by a swelling mechanism, since the unmodified cellulose is hydrophilic and forms a barrier that slows its diffusion [25,27,32]. CC, RC, and OC types of cellulose exhibit this behavior, delaying the drug release during the first 24 h. However, the release profile does not follow a zero order, suggesting the presence of interactions that could generate an irregular release [27]. This effect could be explained by possible delamination, a common problem in multilayer tablets that can misalign the layers and, consequently, affect the drug release [26,32]. Figure 8a shows that the BL-OC tablet releases a higher amount of ibuprofen during the first 6 h, but its subsequent release profile then resembles that of the other formulations. This indicates that bilayer tablets can modulate the drug release rate, albeit with a smaller effect compared to trilayer tablets (Figure 8b).

Regarding the trilayer tablets (TL), cellulose exhibits a more controlled behavior, with release profiles close to the zero order, that is, a linear and sustained release. Zero-order drug release refers to a controlled drug delivery system where the drug is released at a constant rate over time, regardless of its concentration [64]. There are numerous benefits of achieving a zero-order release in tablets for medication, including constant drug levels, improved patient compliance, and a better suitability for drugs with short half-lives [65]. Zero-order drug release is especially useful for conditions like hypertension, diabetes, and pain management, where maintaining consistent drug levels is crucial [66].

As seen in Figure 5, Figure 6 and Figure 7, the ibuprofen release from cellulose-based trilayer tablets is low during the initial hours as it does not exceed 20% and, importantly, follows a stable and predictable trend over time to reach a ~70% release but without plateauing after 120 h, which suggests that a 100% release might be achieved at day 6 following the administration. Combined with an extended release period, a zero-order release is considered optimal, as it maintains a constant drug concentration in the body, thus prolonging its therapeutic effect [23,34,67]. Consequently, it reduces the need for multiple intakes, thereby enhancing the drug therapeutic efficacy and improving patients’ compliance while lowering the risk of adverse effects. Figure 8b shows no significant differences in the release profile between the different types of cellulose utilized in the trilayer tablets. This suggests that the cellulose origin does not directly influence the drug release, but its distribution within the tablet does.

The fact that the cellulose used did not undergo any additional treatments after the extraction explains its strong affinity for water. This suggests that its swelling capacity could influence its ibuprofen release rate.

Taken together, these findings suggest that the rice and orange residue-derived cellulose may be more suitable for applications requiring a highly ordered structure. Both samples have proven to be adequate in the present study, specifically in the formulation of trilayer tablets for sustained drug release. However, ibuprofen was used in the present study as a model drug to investigate the effect of multilayer systems. Future work should focus on the release kinetics of other molecules formulated as drug tablets that are impacted by water and pH solubility.

## 5. Conclusions

The use of biopolymers, such as cellulose, was found to significantly influence the release profile of ibuprofen when incorporated into multilayer tablets. Cellulose derived from agricultural wastes, such as orange peels (OC) and rice husks (RC), represents a promising biomaterial for controlled-release ibuprofen formulations, potentially reducing adverse effects and benefiting patients with chronic diseases by enhancing drug efficacy while decreasing the medication frequency and dosage and improving patients’ compliance. Characterization results indicate that the cellulose extracted from the RC and OC is physically and chemically equivalent to commercial cellulose (CC), validating its suitability for multilayer tablet formulations. Furthermore, multilayer tablets demonstrate a significantly improved and sustained drug release compared to conventional tablets, highlighting their potential in enhanced pharmaceutical delivery.

This study demonstrates the feasibility of using cellulose extracted from plant waste in drug delivery systems, requiring fewer resources and simplifying tablet formulation protocols. High temperatures and pressures enable the successful compression of multilayer ibuprofen tablets exhibiting zero-order controlled release profiles, particularly evident in the three-layer (TL) tablets.

## Figures and Tables

**Table 1:** Tablet formulation according to cellulose type in milligrams (mg).

Tablet Type	Cellulose Type	Ibuprofen (mg)	Cellulose (mg)
Cellulose (C)	CC	-	900
	OC	-	900
	RC	-	900
Compressed commercial ibuprofen (IB)	CC	900	-
	OC	900	-
	RC	900	-
Cellulose mixed with ibuprofen (MIX)	CC	450	450
	OC	450	450
	RC	450	450
Bilayer (BL)	CC	450	450
	OC	450	450
	RC	450	450
Trilayer (TL)	CC	450	225 T 225 B
	OC	450	225 T 225 B
	RC	450	225 T 225 B

T: top layer and B: bottom layer. Composition of tablets for each cellulose source: Commercial Cellulose (CC), Orange Cellulose (OC), Rice Cellulose (RC).

**Table 2:** Temperature and pressure used for tablet compression.

Tablet Type	Temperature (°C)	Pressure (MPa)	Number of Compressions
Cellulose (C)	180	25	1
Compressed commercial ibuprofen (IB)	150	20	1
Cellulose mixed with ibuprofen (MIX)	150	25	1
Bilayer (BL)	180 150	25	2
Trilayer (TL)	180 150 160	20 20 25	3

**Table 3:** Hardness of cellulose tablets compared with commercial ibuprofen tablets.

Cellulose	Tablet Type	Average Hardness Units (HD)
Commercial ibuprofen	Commercial Tablet	59.40
Commercial cellulose	MIX	60.00
	BL	58.50
	TL	59.40
Rice cellulose	MIX	61.30
	BL	60.90
	TL	61.30
Orange cellulose	MIX	61.90
	BL	59.70
	TL	60.30

MIX: ibuprofen mixed with cellulose in equivalent parts, BL: bilayer tablets, and TL: trilayer tablets.

## Data Availability

All data are available in the manuscript or by contacting the corresponding authors.
